# Aberrant Post-Transcriptional Regulation of Protein Expression in the Development of Chronic Obstructive Pulmonary Disease

**DOI:** 10.3390/ijms222111963

**Published:** 2021-11-04

**Authors:** Noof Aloufi, Aeshah Alluli, David H. Eidelman, Carolyn J. Baglole

**Affiliations:** 1 Department of Pathology, McGill University, Montreal, QC H3A 2B4, Canada; Noof.Aloufi@mail.mcgill.ca (N.A.); Aeshah.alluli@mail.mcgill.ca (A.A.); 2 Department of Medical Laboratory Technology, Applied Medical Science, Taibah University, Universities Road, Medina P.O. Box 344, Saudi Arabia; 3 Department of Medicine, McGill University, Montreal, QC H4A 3J1, Canada; david.h.eidelman@mcgill.ca; 4 Department of Pharmacology and Therapeutics, McGill University, Montreal, QC H3G 1Y6, Canada

**Keywords:** post-transcriptional regulation, RNA binding proteins, miRNAs, COPD

## Abstract

Chronic obstructive pulmonary disease (COPD) is an incurable and prevalent respiratory disorder that is characterized by chronic inflammation and emphysema. COPD is primarily caused by cigarette smoke (CS). CS alters numerous cellular processes, including the post-transcriptional regulation of mRNAs. The identification of RNA-binding proteins (RBPs), microRNAs (miRNAs), and long non-coding RNAs (lncRNAs) as main factors engaged in the regulation of RNA biology opens the door to understanding their role in coordinating physiological cellular processes. Dysregulation of post-transcriptional regulation by foreign particles in CS may lead to the development of diseases such as COPD. Here we review current knowledge about post-transcriptional events that may be involved in the pathogenesis of COPD.

## 1. Introduction

Human organ systems rely on the dynamics of gene expression to regulate homeostasis, cell survival, fate and differentiation, as well as responses to stress and environmental signals [1]. Eukaryotic cells have developed sophisticated mechanisms to produce and use the transcripts with optimum efficacy through their life cycle. When RNA is synthesized in the nucleus, its biogenesis, translocation to the cytosol, and interaction with proteins and other components are necessary to achieve their encoding function. All these steps undergo post-transcriptional regulation of that initial messenger RNA (mRNA), and these steps comprise an important part of overall gene and protein expression. Post-transcriptional regulation is a coordinated process that takes place when the RNA is transcribed, but before it is translated into protein. Factors that associate with and regulate target mRNAs at the post-transcriptional level are RNA-binding proteins (RBPs), microRNA (miRNA), and long non-coding RNA (lncRNA) [2,3]. In mammalian cells, the fate of mRNA is controlled by almost 2000 RBPs [4] and ~2300 miRNAs [5]. In humans, there are approximately 172,216 lncRNAs [6], and 27,919 lncRNAs have been identified in a variety of human primary cells [7]. These factors dynamically modulate mRNAs during biological processes, and their dysregulation is likely to be involved in pathological processes. Cigarette smoke (CS) causes a variety of chronic lung disorders, including chronic obstructive pulmonary disease (COPD) and lung cancer. CS is responsible for approximately 70% of COPD cases [8] and 90% of lung cancer cases [9], and remains a major cause of morbidity and mortality worldwide. The pathogenesis of diseases associated with smoking involves the dysregulation of numerous cellular and physiological pathways, such as proliferation, apoptosis, and inflammation [9,10,11,12,13,14]. These processes are controlled at the post-transcriptional level via the regulation of mRNAs. In this review, we will discuss RBPs, miRNAs, and lncRNAs that regulate the post-transcriptional modifications of mRNAs, and their involvement in normal physiology. We will then highlight post-transcriptional regulatory mechanisms that are dysregulated in response to smoke, and, thus, may be implicated in the pathogenesis of COPD.

## 2. Post-Transcriptional Regulation of mRNAs

### 2.1. RNA Binding Proteins (RBPs)

RBPs are a group of over 2000 proteins, each possessing multiple RNA binding domains, and which are known to be involved in RNA decay [4]. RBPs associate with RNA transcripts and form ribonucleoprotein (RNP) complexes after transcription. Some RBPs bind early during RNA synthesis to precursor mRNA (pre-mRNA) and remain bound to the pre-mRNA until its degradation or translation, whereas other RBPs recognize and bind to pre-mRNA for specific processes, such as splicing, stability, transport, and cellular localization [15]. The diversity of RBP functions suggest that several RNA-binding domains (RBD) are responsible for RNA recognition and for recruitment to specific RNA targets [16,17]. RBPs contain one or multiple RNA-binding domains, such as the RNA-recognition motif (RRM), K-homology domain (KH), double-stranded RBD (dsRBD), zinc fingers (Znf), DEAD box helicase domain, among others. There is diversity in the specificity and affinity of RBD interaction with RNA [4,18,19]. Some RBPs with dsRBD interact with the phosphate-sugar backbone of their RNA targets [4,18]. Other RBPs, such as those with RRMs, interact in a sequence-specific manner with the nucleotide base and shape complementarity of the RNA [4,18,19,20]. In this section, we will explore the role of RBPs in different aspects of RNA biology.

#### 2.1.1. Biological Functions of RBPs

**Figure 1:**
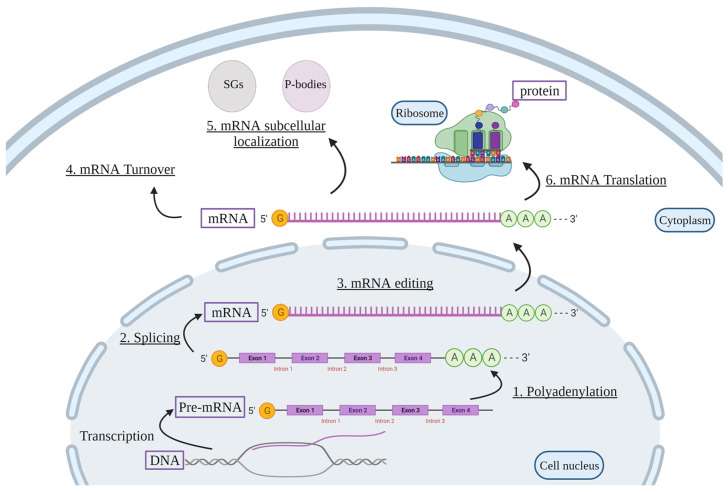
**Cellular functions of RBPs.** RBPs are involved in post-transcriptional regulation of target mRNAs. Pre-mRNA is first transcribed from the DNA. Then, RBPs regulate the production of mature mRNA via polyadenylation (**1**), splicing (**2**), and mRNA editing (**3**). RBPs can also regulate mRNA stability (**4**) and mRNA subcellular localization (**5**) within the cell in SGs or P-bodies, as well as mRNA translation into proteins (**6**). Pre-mRNA, precursor mRNA; SGs, stress granules; P-bodies, processing-bodies. Created with BioRender.com.

RBP-mediated post-transcriptional regulation is essential for proper cellular function, and its perturbation can lead to the development of disease. For example, the fragile X syndrome of mental retardation is caused by a defect in the RBP fragile X mental retardation protein, which is important for normal brain development [21]. The fate of RNA, from transcription to translation, is highly dependent on RBP-mediated polyadenylation, pre-mRNA splicing, as well as mRNA editing, turnover, subcellular localization, and translation (Figure 1).

##### Polyadenylation

Polyadenylation of pre-mRNA is an essential processing event for RNA nuclear export, stability, and translation. Polyadenylation is a maturation step in which all pre-mRNAs in eukaryotic cells, except mRNA encoding histones, receive poly(A) tails of around 200 adenine (A) nucleotides to their 3’ end by a multiprotein machinery complex [22,23]. This occurs in a coupled cleavage reaction whereby the pre-mRNA is first cleaved between AAUAAA sequences upstream and U/GU rich sequences downstream of the cleavage site, followed by the addition of a polyadenosine tail. The cleavage and polyadenylation machinery consists of four multi-subunit protein complexes: the cleavage and polyadenylation specificity factor (CPSF); the cleavage stimulation factor (CstF); and mammalian cleavage factors I and II (CFIm and CFIIm) [24]. The CPSF protein complex consists of six protein subunits that are vital for cleavage of pre-mRNA and interaction with AAUAAA sequences [23,24]. CstF consists of three subunits that interact with the downstream element and upstream site of the pre-mRNA [22,24,25]. CFIm and CFIIm are required for the cleavage step [25,26]. Then, poly (A) polymerase, stimulated by CPSF and the RBP nuclear poly(A) binding protein (PABPN1), adds the poly(A) tail to the cleavage product of the synthesized pre-mRNA molecule to produce mature mRNA [27,28]. The best-characterized mRNA-stabilizing factor, hu antigen R (HuR)/embryonic lethal abnormal vision *Drosophila*-like (ELAVL1), is also involved in the polyadenylation step. HuR is a ubiquitously-expressed RBP which is predominantly nuclear, but shuttles between the nucleus and cytoplasm. HuR blocks polyadenylation of the simian virus 40 late (SVL) poly(A) site that has U-rich sequences both upstream and downstream of the cleavage site. This leads to a decrease of SVL poly(A) site-containing mRNA, and an increase of pre-mRNA levels [29]. 

##### Pre-mRNA Splicing

Splicing of pre-mRNA is a step of gene expression in which introns (noncoding sequences) are removed, and exons (coding sequences) are assembled by the spliceosome. The spliceosome is a ribonucleoprotein complex composed of five small nuclear RNAs (snRNAs), U1, U2, U4, U5, and U6, and more than 50 protein factors, such as U2 auxiliary factor and SR (serine-arginine rich) proteins [30,31]. Some exons are constitutively spliced [32]. However, many exons are alternatively spliced, in which more than one mRNA can be generated from a single pre-mRNA. At least 74% of human multi-exon genes express several mRNAs through alternative splicing (AS) [33]. Studies using high-throughput sequencing showed that ~95% of multi-exon genes undergo AS [34,35]. RBPs also regulate this process, including SR proteins and heterogeneous nuclear ribonucleoproteins (hnRNPs) [36]. In human cells, hnRNPs are the most abundant RBPs that regulate AS of pre-mRNAs. Genome-wide analysis showed that more than half of all AS events are regulated by six major hnRNP proteins: A1; A2/B1; H1; F; M; and U [37]. One of the RBPs involved in this process is HuR, which can promote exon 6 skipping of the apoptosis receptor Fas pre-mRNA through the inhibition of the U2 auxiliary factor 65 kDa association with the upstream 3’ splice site—this leads to the production of the soluble isoform of Fas that prevents apoptosis [38]. 

##### mRNA Editing

RNA editing is a type of RNA modification characterized by the alteration of site-specific RNA sequences from that encoded in DNA [39]. The RNA codon and protein sequence are changed if the editing occurs in the coding region [40]. When editing occurs in the noncoding regions, it may affect splicing, stability, or translation of the mRNA [41,42]. RNA editing, mediated by adenosine deaminases acting on RNA (ADAR) proteins, involves adenosine (A) deamination to inosine (I) that is then recognized as guanosine by the translational apparatus [39,41,43,44]. ADARs contain two or three RNA binding domains (RBDs), and a highly conserved deaminase domain [45]. Three ADAR proteins, ADAR1, ADAR2, and ADAR3, are present in humans [44,45,46,47]. A-to-I editing can occur in noncoding regions of the RNA, in Alu repeats, which are good substrates for ADAR proteins [42,48,49].

##### mRNA Turnover

The translation of mRNA is coupled with its stability and decay. RBPs that regulate mRNA stability are either mRNA decay activators or mRNA stabilizers. Activators of mRNA decay recognize the constitutive decay AU- and GU-rich elements of their target mRNAs, and affect its cellular levels by several mechanisms [50]. For example, tristetraprolin (TTP), also known as zinc finger protein 36, is an RBP that promotes mRNA decay [51]. TTP promotes deadenylation of tumor necrosis factor alpha (TNF-α) mRNA and its degradation upon exposure to lipopolysaccharide (LPS) [52,53,54]. TTP also downregulates numerous inflammatory mRNAs, such as interleukin (IL)-6, IL-2, and cyclooxygenase-2 (COX-2/ PTGS2) [55,56,57]. Other RNA-binding proteins implicated in mRNA decay include KH-type splicing regulatory protein (KSRP) [58], Roquin [59], and ARE/poly(U)-binding/degradation factor 1 (AUF1) [60,61,62]. Conversely, other RBPs act as mRNA stabilizers, and impede mRNA degradation. One of the best known RBPs with a regulatory influence on mRNA stability is HuR. Besides HuR regulation of pre-mRNA polyadenylation and splicing, HuR also targets mRNAs which have U- or AU-rich elements (AREs) in the 3’-untranslated region (UTR)—these mRNA typically encode proteins involved in cell proliferation, differentiation, migration, apoptosis, inflammation, and fibrosis [63,64,65,66,67,68,69,70]. 

##### mRNA Subcellular Localization

Localization of mRNA is critical for protein synthesis. Stress granules (SGs) and processing (P-) bodies are cytoplasmic RNA granules consisting of aggregates of ribonucleoprotein complexes. SGs and P-bodies are assembled in stressed and in unstressed cells, respectively [71]. SGs sequester mRNAs for storage and translational silencing [72]. In this context, HuR also regulates the subcellular localization of mRNAs. In human osteoarthritis chondrocytes, for example, in response to IL-1β, *PTGS2* mRNA is sequestered in SGs by HuR, thereby decreasing protein levels due to a delay in translation [73]. SGs also contain other RPBs involved in RNA metabolism, including poly(A)-binding protein (PABP), T-cell intracellular antigen 1 (TIA-1), and TTP [74]. In contrast, P-bodies contain mRNAs targeted for degradation, and the RBPs are involved in this process [75]. For instance, Roquin suppresses inducible co-stimulator (ICOS) expression, which prevents autoimmunity through its association with P-bodies in T-helper cells [76]. 

##### mRNA Translation

The regulation of mRNA translation controls gene expression in the cytoplasm. Numerous proteins, including RBPs, regulate mRNA location and assembly into ribosomes for protein synthesis. For example, the RBP PABP that binds to stable mRNA also interacts with eukaryotic initiation factors 4E (eIF4E), whereby the 48S and 80S ribosome initiation complex are assembled and translation is initiated [77]. Another example is the RBP TIA-1, which represses translation of various mRNAs, including *PTGS2* mRNA [78]. Some RBPs can inhibit or promote translation depending on the context. HuR promotes the translation of prothymosin α, an enhancer of cell survival, in response to irradiation [79], but can inhibit the translation of p27, a cyclin-dependent kinase inhibitor, and Wnt5a, a non-transforming Wnt protein, during proliferation and oncogenesis, respectively [80,81]. 

### 2.2. miRNAs

miRNA are small non-coding RNAs (~22 nucleotides) that affect gene expression [82,83]. In the nucleus, miRNAs are synthesized from primary miRNA which are processed to precursor miRNA (pre-miRNA) by ribonuclease III (RNase III), Drosha, and the RBP DiGeorge syndrome critical region 8 (DGCR8). The resulting pre-miRNA is exported to the cytoplasm by the nuclear transport factor exportin-5. The pre-miRNA is then further processed to ~22-nt miRNA by Dicer, another RNase III enzyme [83,84]. miRNAs pair to the 3′ UTR of mRNA by partial sequence matching after being incorporated into the RNA-induced silencing complex (RISC). This leads to direct post-transcriptional repression by inhibiting translation and/or inducing mRNA decay [85,86]. In 1993, lin-4, a developmental regulator, was the first miRNA identified in *Caenorhabditis elegans* [87,88]. At the present time, about 2300 miRNAs have been discovered in humans, around half of which are annotated in miRbase [5]. The importance of the RNA interference (RNAi) machinery in mammals is highlighted in studies where Dicer or DGCR8 were knocked out, leading to embryonic lethality [89,90].

#### Biological Function of miRNAs

The function of miRNAs has been explored in murine knockout models, which have revealed important roles for miRNA in various biological processes, including development and immunity. For example, targeted ablation of miRNA-1-2, a muscle-specific miRNA, leads to cardiac morphogenetic and electrophysiologic defects [91]. Furthermore, the knockout of miRNA-155 causes defects in adaptive immunity [92]. miR-223 is a myeloid-specific miRNA that targets Mef2c, a transcription factor which promotes myeloid progenitor proliferation. miR-223 null mice have marked neutrophilia, and, consequently, develop pulmonary inflammation and exaggerated tissue destruction in response to LPS [93]. Some miRNAs have multiple essential functions. An example is miR-17~92: these knockout mice die postnatally with heart defects and lung hypoplasia, but also exhibit defects in B cell development [94]. 

### 2.3. LncRNA

LncRNA are a class of ncRNA that are more than 200 nucleotides in length and do not translate into protein [95,96,97]. lncRNAs can be classified based on their genomic proximity to protein-coding genes [97,98]. lncRNA are sub-grouped into five main classes, including: (1) antisense lncRNAs or natural antisense transcripts (NATs); (2) sense lncRNAs; (3) intronic lncRNAs; (4) long intergenic RNAs (lincRNAs); and (5) bidirectional lncRNAs [97,98]. The most common classes of lncRNAs in humans are antisense and intergenic lncRNAs [98]. 

#### Biological Function of lncRNA

Based on the molecular mechanisms of action, lncRNA can also be categorized as decoy, scaffold, and guide [97,99]. Decoy lncRNA bind and capture different molecules, including proteins, transcription factors, and other regulatory RNA, which results in inhibition of functions [97,99]. Decoy lncRNA can positively or negatively affect transcription [97,99]. Decoy lncRNA can titrate and prevent transcription factors or repressors from binding their target gene promoters [97,99]. Scaffold lncRNA have binding sites that interact with distinct effector molecules, and, thus, can serve as a platform for connecting various protein complexes [97,99]. lncRNA can also guide chromatin to specific genomic regions to regulate gene expression [100,101,102], increase DNA methylation by binding with DNA methyltransferases [103], participate in AS by directly interacting with splicing factors or proteins [104], or directly interact with either RNA polymerase II (Pol II) or transcription factors [100,101,105]. lncRNA can also regulate post-transcriptional events [100,101,105] to control mRNA function by changing its stability, splicing patterns, and translation [105]. lncRNA can alter mRNA stability by interacting with a specific sequence motif of an RBP, resulting in the formation of lncRNA-protein complexes [106]. As such, lncRNA have numerous cellular functions including regulating proliferation, differentiation, and survival.

## 3. Post-Transcriptional Regulation in COPD

### 3.1. COPD Pathogenesis

COPD is a leading cause of chronic morbidity and mortality worldwide [107]. The World Health Organization (WHO) lists COPD as the third leading cause of death [108], with its prevalence expected to increase by more than 30% in the coming decade. The Global Initiative for Chronic Obstructive Lung Disease (GOLD) defines COPD as a lung disease characterized by progressive and irreversible airflow limitation, which is usually associated with an abnormal inflammatory response in the airways and lungs to noxious particles or gases. The clinical presentation in COPD patients includes cough, sputum production, and/or dyspnea [109]. Chronic airflow limitation is due to both emphysema, which is the irreversible destruction of the gas-exchanging alveoli, and chronic bronchitis, a disease entity characterized by the presence of a productive cough for at least three consecutive months during the last two consecutive years [110]. The risk factors for the development of COPD include a combination of genetic susceptibility and exposure to environmental toxicants [111]. 

The main cause of COPD is CS [111]. Globally, there are around 1.3 billion tobacco smokers [112]. CS is a complex combination of thousands of chemicals (approximately 7000 individual components) of which at least 158 have known toxicological properties [113,114]. The components with the strongest correlations to disease development are polycyclic aromatic hydrocarbons (PAHs) and N-nitrosamines. Other components that are associated with pulmonary toxicity include free radicals, catechols, and aldehydes [9]. Beyond CS, additional risk factors for COPD include childhood asthma and respiratory infections, exposure to ambient and biomass air pollution, exposure to second-hand smoke, and occupational exposure to dust and fumes [8,110,111,115]. Genetic factors are associated with the development of COPD. The most notable of these is deficiency of alpha-1 antitrypsin (α1AT), which accounts for approximately 1–2% of COPD cases [111,115]. Clinical symptoms can develop in patients many years after starting smoking, with COPD commonly diagnosed in people over the age of 50 years, with the highest frequency at approximately 70 years [116]. 

**Figure 2:**
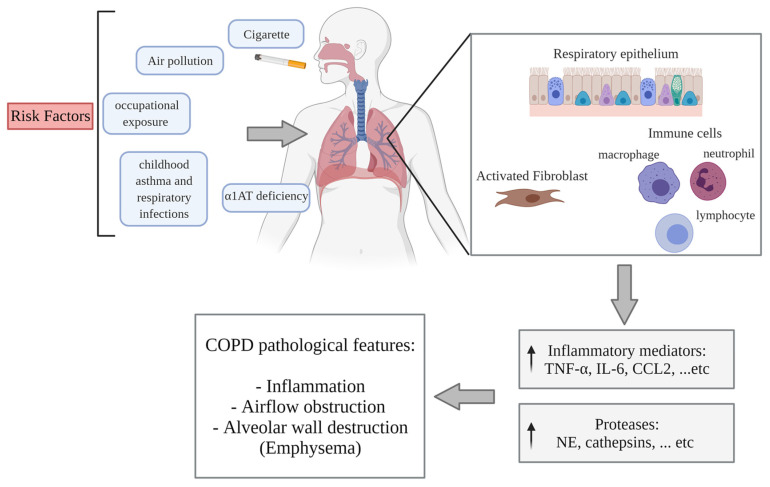
**Overview of the etiology and pathogenesis of COPD.** Risk factors for the development of COPD include: cigarette smoke; air pollution; occupational exposures; childhood asthma; respiratory infections; and alpha 1-anti-trypsin (α1AT) deficiency. Upon exposure to inhaled toxicants, lung structural cells, including epithelial cells and fibroblasts, as well as alveolar macrophages, are activated. These cells produce inflammatory mediators to recruit additional inflammatory cells, such as neutrophils, macrophages, and lymphocytes, to the site of exposure. This augments the expression of inflammatory mediators, such as TNF-α, IL-6, CCL2, CCL7, CXCL1, CXCL5, CXCL8 (IL-8), LTB4, and COX-2, and releases proteases, such as NE, cathepsins, and MMPs. This cascade of events can lead to chronic pulmonary inflammation, airflow obstruction, and alveolar wall destruction (emphysema) in a susceptible individual. Created with BioRender.com.

Mechanistically, the development of COPD is initiated by inflammation caused by repeated exposure to CS, which induces a pulmonary inflammatory response in several cell types, including epithelial cells, fibroblasts, and macrophages [10,11,12]. Repeated exposure to CS leads to the additional recruitment of innate and adaptive immune cells, including neutrophils, macrophages, and lymphocytes. This, in turn, amplifies the expression of inflammatory mediators, such as TNF-α, IL-6, C-C motif ligand 2 (CCL2), CCL7, C-X-C motif ligand 1 (CXCL1), CXCL5, CXCL8 (IL-8), leukotriene (LT) B4, and COX-2 [11,117,118,119,120,121,122]. In addition to inflammation, other pathogenic mechanisms involved in COPD include an imbalance between proteases and antiproteases, as well as heightened oxidative stress in the lungs [123]. Repeated exposure to CS causes the release of proteases. The increased production of lung proteases, such as neutrophil elastase (NE), and the resulting apoptosis of alveolar septal cells lead to the destruction of alveolar walls, causing emphysema [124]. Moreover, proteases, such as NE, cathepsin G, and proteinase-3 promote mucus secretion by increasing the number of goblet cells, stimulating degranulation in these cells, and causing the enlargement of submucosal glands. The combination of mucus hypersecretion, inflammation in the airway walls and lumen, fibrosis formation around the small airways, and loss of lung elastic recoil leads to the narrowing of these airways, leading to airflow obstruction [118,124,125] (Figure 2). Finally, the inflammation in COPD further increases during acute exacerbations, which are defined as a worsening of day-to-day symptoms, and are predominantly caused by bacterial or viral infection [117]. Exacerbations in COPD are strongly correlated with an increase in hospitalization and mortality, and a decrease in lung function [116].

No disease-modifying therapies exist for COPD. After smoke exposure and in COPD, transcriptional regulation alters the expression of inflammatory mediators, such as IL-6, COX-2, TNF-α, IL-1β, and IL-8 [126]. However, post-transcriptional regulation of mRNA has emerged as an important factor in the overall regulation of gene and protein expression in response to environmental exposures. A better understanding of the mechanistic underpinnings of post-transcriptional regulation of mRNA could lead to the development of new targeted therapies for COPD. Here, we summarize the current state-of-knowledge of post-transcriptional regulation that is applicable to pathogenic mechanisms implicated in the response to CS and in the development of COPD. 

### 3.2. RBPs 

#### 3.2.1. The Response of RBPs to CS

RBPs appear to play a role in the cellular response to CS. We have previously shown that lung fibroblasts produce COX-2 in response to CS [11,121], and that the aryl hydrocarbon receptor (AhR) destabilizes *PTGS2* mRNA by preventing HuR translocation into the cytoplasm [127]. It is now well-described that COX-2, and other inflammatory mediators that are increased in COPD [11,117,118,119,120,121,122], are regulated by HuR [128]. Furthermore, HuR controls IL-8 secretion from human bronchial epithelial cells exposed to cigarette smoke extract (CSE) in combination with human rhinovirus (HRV) [129]. HRV infection is a common trigger of virus-associated COPD exacerbations that are correlated with persistent lung inflammation [130,131]. Thus, HuR may be involved in the early pathogenic events, such as inflammation, that are associated with the development of COPD and/or exacerbations.

Another RBP that has been studied in the context of smoking is RNA-binding motif protein 5 (RBM5). The gene of RBM5, also known as H37 or Luca15, located in chromosomal region 3p21.3, is frequently deleted in heavy smokers and lung cancer patients [132,133]. *Rbm5* loss-of-function (heterozygous) mice exposed to the tobacco carcinogen 4-(methylnitrosamino)-1-(3-pyridyl)-1-butanone (NNK) develop more aggressive lung cancer [134]. In cells exposed to CSE, RBM5 mRNA and protein levels are decreased, and β-catenin is increased. β-catenin is a key player in canonical Wnt signaling, whose activation induces genes involved in cell differentiation [135]. β-catenin is increased in proximal airway epithelium in COPD, with activation of Wnt/β-catenin signaling increasing epithelial-to-mesenchymal transition (EMT) [136]. EMT is a process where epithelial cells gradually lose cellular polarity and adhesiveness, and acquire migratory capacity and invasiveness, like that in a mesenchymal phenotype. EMT is increased in bronchial epithelial cells from COPD patients, which contributes to fibrosis formation around the small airways, leading to airflow obstruction [137]. Although the evidence is indirect, these studies raise the prospect that RBM5 could regulate EMT in COPD through the β-catenin pathway. At this writing, the expression and function of RBM5 in COPD is unknown.

#### 3.2.2. The Regulation of RBPs in COPD

Elucidation of changes in the expression and the function of RBPs may suggest putative pathogenetic roles for them. Targeting RBPs could also be a novel therapeutic strategy. However, only a handful of studies directly investigate RBPs in COPD. One of these was a genome-wide association study which identified iron-responsive element binding protein 2 (IRP2 or IREB2), an RNA-binding protein that regulates cellular iron homeostasis, as a COPD susceptibility gene. *IRP2* mRNA and protein levels are elevated in lungs from COPD subjects [138,139,140,141], and IRP2 expression is increased in the lungs of mice chronically exposed to CS. Furthermore, the knockout of IRP2 protected mice from CS-induced pulmonary inflammation and impairment of airway mucociliary clearance. Mechanistically, IRP2 in the lungs induces mitochondrial dysfunction by promoting mitochondrial iron loading and cytochrome c oxidase [142]. Previous observations have shown that iron deposition is increased in lungs from severe COPD patients, as well as in response to CS [143,144], which may be regulated by the elevation of IRP2.

Another RBP studied in the context of COPD is HuR, where its expression is increased in the airway epithelium from smokers with or without COPD [145]. This suggests that this increase is likely due to smoking, a notion further supported by a separate study showing that HuR expression is similar in the bronchial epithelium from both COPD subjects and smokers without COPD [146]. Mechanistically, recent studies support a role for HuR in the pathogenesis of COPD. For example, HuR stabilizes zinc finger E-box binding homeobox 1 (ZEB-1), a transcription factor involved in EMT. The expression of ZEB-1 is increased in the airway epithelium from COPD [145]. This suggests the possibility that HuR may be involved in the pathogenesis of COPD by regulating EMT.

Finally, the RBP AUF1, which participates in mRNA decay, is decreased in the bronchial epithelium from COPD subjects compared to smokers without COPD. Analysis of a microarray from a primary airway epithelium of COPD revealed that AUF1 target genes are upregulated, including those associated with inflammation [146]. Although this suggests that AUF1 may regulate the expression of inflammatory genes involved in COPD, direct regulation by AUF1 of these downstream mRNA, and its implications for the pathogenesis of COPD, remain to be investigated. Recently, a mapping profile of RBPs indicated that most RBP genes are downregulated in the small airway epithelium of those with COPD, comparing to non-smokers and smokers [147]. Overall, these studies raise the possibility that RBPs may be involved in the development of COPD. As little is known about the direct role of RBPs in COPD per se, below we summarize studies which have examined related mechanisms associated with the development of this disease. 

##### RBPs in Inflammation

CS causes direct damage to airway and alveolar epithelial cells, which leads to the recruitment of inflammatory cells, and the release of numerous inflammatory mediators, including TNF-α [9,11,117,118,119,120,121,148], the overexpression of which induces pulmonary inflammation and airspace enlargement [149]. An RBP known to regulate TNF-α expression is TTP. TTP is generally an anti-inflammatory RBP, as TTP knockout mice have a proinflammatory phenotype [150]. TTP promotes mRNA decay of *TNF-α* by binding to AREs present within the 3’ UTR [52]. TTP also destabilizes other mRNAs associated with inflammation, including *PTGS2*, *IL-6*, *CXCL8,* and *CCL2* [151,152]. Glucocorticoids, which are used clinically in COPD, elevate mRNA and protein levels of TTP that are crucial for glucocorticoid-mediated inhibition of *TNF-α* mRNA [153]. Glucocorticoid inhibition of other inflammatory genes, including *CCL2*, *CCL7*, *CXCL1,* and *CXCL5,* is also abrogated in TTP-knockout cells [154]. Overall, these studies suggest that TTP target mRNAs encode proteins responsible for the inflammatory response associated with COPD. 

AUF1 is another RBP that induces the decay of target mRNAs, including *TNF-α*. AUF1 knockout mice are susceptible to endotoxin challenge due to TNF-α and IL-1β overproduction [155]—these mice also exhibit chronic dermatitis with age, concomitant with TNF-α and IL-1β overexpression [156]. Given that AUF1 expression is decreased in COPD [146], and that many inflammatory mediators regulated by AUF1 are also upregulated in COPD, it is possible that dysregulation of AUF1 may contribute to the inflammatory response associated with this disease.

While AUF1 and TTP may be important in controlling inflammation by promoting mRNA decay, other RBPs, such as HuR, stabilize mRNA associated with inflammation, such as *CCL2* and *TNF-α*, in pulmonary epithelial cells [69], as well as macrophages [157]. HuR itself is also activated by TNF-α, as evidenced by its translocation to the cytoplasm following treatment with TNF-α and IL-4 to regulate *CCL-11* (eotaxin) mRNA levels [158]. This may be relevant in the context of COPD, as eotaxin is an inflammatory chemokine whose expression is elevated in blood from COPD patients [159]. Thus, these studies highlight HuR as a key player in post-transcriptional gene regulation of inflammatory mRNAs.

##### RBPs in Apoptosis and Protease Expression

Emphysema is characterized by the loss of lung structural cells, including alveolar epithelial cells and fibroblasts [109,160]. Mechanistically, emphysema is thought to develop because of CS-induced apoptotic cell death [13,14]. Evidence for this comes from studies where intra-tracheal administration of active caspase-3 induced epithelial cell apoptosis, elastolytic activity in bronchoalveolar lavage (BAL), and airspace enlargement in mouse lungs [161]. Other proteins, such as vascular endothelial growth factor (VEGF), help alveolar cells withstand damage by CS. Experimentally, the blocking of VEGF receptors stimulates apoptosis of alveolar cells, and induces an emphysema-like pathology [162,163]. In COPD, the level of VEGF is decreased, which may be a contributing factor to the development of emphysema in people [164]. Many of the RPBs mentioned above that regulate inflammation also have roles in apoptosis. For example, TTP destabilizes *VEGF* mRNA [165]. In contrast, hnRNP L stabilizes *VEGF* expression [166]. hnRNP L is a multifunctional splicing factor that is involved in the regulation of alternate splicing and mRNA stability [166,167]. Interestingly, the knockout of hnRNP L in hematopoietic stem cells causes cell death through caspase-dependent pathways [168], raising the possibility that the downregulation of VEGF and upregulation of cell death in COPD could be regulated by hnRNP L.

In addition to aberrant cell death, lung tissue destruction in COPD is mediated by proteases. Activated neutrophils are a potent source of proteases, such as NE, cathepsin G, proteinase-3, MMP-8, and MMP-9, all of which can contribute to the destruction of alveolar walls [169,170]. Macrophages also secrete MMP-9 and MMP-12, as well as cathepsins L and S [171]. RBPs implicated in the regulation of proteases include TTP, which destabilizes *MMP-9* and *MMP-2* mRNAs [172], and HuR, which binds to *MMP-9* mRNA to stabilize its expression [173]. Changes in the expression of RBPs can greatly impact their function and, thus, regulation of protease expression. For example, nitric oxide and IL-10 can both reduce HuR expression and its subsequent binding to *MMP-9* mRNA [174,175].

#### 3.2.3. Interplay of RBPs

Dynamic interactions between RBPs may fine-tune post-transcriptional modifications of common mRNAs. RBPs can either cooperate or compete to bind target mRNAs. For example, ADAR1 cooperates with HuR, and forms an RNA-dependent complex, which regulates the stability of ADAR1 targets in human B cells [176]. ADAR1 mediates A-to-I editing of cathepsin S (*CTSS*) [177], a cysteine protease associated with the remodeling/degradation of connective tissue and basement membrane [178]. HuR also binds to the 3′ UTR of *CTSS* mRNA, and controls its stability and expression [177]. Interestingly, CTSS is elevated in smokers and COPD patients [179]. HuR and TIA-1 can also interact to impact mRNA encoding programmed cell death 4 (PDCD4), a tumor suppressor that induces apoptosis. Here, increasing TIA-1 prevents HuR from binding to the *PDCD4* mRNA, whereas decreasing TIA-1 induces HuR binding to the *PDCD4* mRNA [180]. Furthermore, TTP interacts with PABP1 in activated primary mouse bone-marrow-derived-macrophages, and represses the translation of TTP target mRNAs involved in the inflammatory response [181]. Together, these studies illustrate the interplay of RBPs in the regulation of various post-transcriptional processes involved in physiological and pathological mechanisms.

### 3.3. miRNA

Another aspect of post-transcriptional regulatory mechanisms of relevance for smoke-induced lung disease are miRNAs. Thus, miRNAs are being pursued as therapeutic targets or, through their utility, as a diagnostic tool. For example, *let-7*, miR-206, and miR-16 are downregulated in lung cancer. The observation of miR-16 is of interest, as overexpression of miR-16 prevents cell proliferation and invasion [182,183,184,185]. Interestingly, a miR-16 mimic (the TargomiR drug, MesomiR-1) is in a phase 1 trial for patients with malignant pleural mesothelioma and lung cancer. Thus far, this trial has shown safety, as well as initial signs of response [186,187]. These studies suggest that miRNAs play a key role in the progression of diseases, and that targeting miRNAs as a diagnostic or as a therapeutic target for disease is attractive. Numerous studies have now comprehensively interrogated changes in miRNA expression caused by smoking and/or in COPD [188,189,190,191,192,193,194,195,196,197,198,199,200,201,202,203,204,205,206]. Smoking alters miRNA expression, as shown by observations in human smokers, as well as in lungs of mice and rats exposed to CS [190,191]. One such study showed that 34 miRNAs are differentially expressed between never-smokers, smokers, and COPD subjects, including 8 miRNAs that were downregulated when compared with never-smokers [197]. Some miRNAs are altered in the context of COPD itself, and the nature of this dysregulation may be cell-type specific, as observed for miR-199a-5p. Though miR-199a-5p expression is reduced in T-cells from COPD patients [198], miR-199a-5p expression is elevated in lung tissue [199].

As the severity of COPD increases, miRNA expression changes. For example, miR-206, miR-133a-5p, and miR-133a-3p are upregulated in extracellular vesicles of plasma from moderate COPD patients compared to mild and severe patients—these miRNAs are involved in hundreds of biological processes that are associated with COPD features [196]. miRNAs are differentially expressed based on emphysema severity, and these changes correlate with the changes in the expression of their predicted mRNA targets [205]. Some of the miRNAs altered by emphysema severity include miR-34c, miR-34b, miR-133b, and miR-149, which are reduced in lung tissue from COPD patients with moderate emphysema compared to mild disease [206]. Thus, there is differential expression of miRNAs in COPD and/or in response to CS [188,189,190,191,192,193,194,195,196,197,198,199,200,201,202,203,204,205,206], which may, in turn, perturb downstream pathways controlling pathological processes, such as inflammation and cell survival.

#### 3.3.1. miRNA and Pathogenic Mechanisms of COPD

Experimental studies profiling miRNA expression have shed light on the possible biological significance of dysregulated miRNA to COPD pathogenesis, including their ability to regulate smoke-induced inflammation. In one such study of miRNA expression in current and never-smokers’ bronchial airway epithelium, it was found that 28 miRNAs are differentially expressed. The most downregulated miRNA in smokers was miR-218—its expression is also decreased in primary bronchial epithelial cells exposed to cigarette smoke condensate [188]. The mature form of miR-218 is generated from two separate loci, miR-218-1 and miR-218-2 [207]. miR-218 targets MAFG, a transcription factor that is elevated in smokers and in response to CS [188], and induces pro-inflammatory gene transcription [208]. miR-218-5p (miR-218-2) is also significantly downregulated in lung tissue from smokers and COPD patients, as well as in response to experimental smoke exposure. Overexpression of miR-218-5p in normal human bronchial epithelial cells exposed to CSE reduces the mRNA and the protein levels of CCL20 and CXCL8 [200], chemokines that are involved in the pathogenesis of COPD [118,209]. Furthermore, inhibiting miR-218-5p in mice exposed to smoke worsens smoke-induced inflammation [200]. These findings indicate an important role of miR-218-5p in the CS-induced inflammatory response.

Other miRNA implicated in regulating inflammation are miR-181c, miR-145, miR146a, and miR-16, all of which are downregulated in response to CS and/or in COPD [190,201,202,210]. Overexpression of miR-181c in mice exposed to CS reduces neutrophil infiltration, in conjunction with IL-6 and CXCL8 expression in the lungs. Overexpression of miR-181c in primary human bronchial epithelial cells treated with CSE also decreases IL-6 and CXCL8 expression [201]. miR-145 is also downregulated in the lungs of mice exposed to CS [190]. Outside the lungs, miR-145 overexpression represses the release of IL-6 and CXCL8 [211], as well as VEGF and MMP-9 [212]. Finally, miR-146a is downregulated in the serum of COPD patients with acute exacerbations when compared with stable COPD patients and healthy controls, which, in turn, is negatively correlated with inflammatory cytokines [202]. Mechanistically, we found that miR-146a suppresses cigarette smoke-induced COX-2 protein expression in murine lung fibroblasts [203]. miR-146a also suppresses COX-2 in lung fibroblasts from COPD subjects upon IL-1β/TNF-α stimulation, and, therefore, reduces prostaglandin (PG)E2 production [204]. miR-16 is another miRNA whose expression is decreased in lung fibroblasts from heavy smokers and COPD patients compared to those from non-smokers [210]—miR-16 also silences COX-2 [213]. These findings suggest that the loss of this miRNA can eventually enhance COX-2 expression in response to smoke.

Other miRNAs are increased by smoking, include miR-101, miR-144 [192], miR-135b [193,194], and miR-223. miR-223 leads to a decrease in histone deacetylase 2 (HDAC2) expression, which alters the expression of pro-inflammatory chemokines [195]. miR-101 and miR-144 are higher in human bronchial epithelial cells exposed to CS, and suppresses expression of the cystic fibrosis transmembrane regulator (CFTR), a chloride channel which maintains airway surface fluid homeostasis [192]. miR-101 is also upregulated in lungs of mice exposed to CS [192].

Alveolar macrophages are among the first cell types to respond to smoke inhalation, as these innate immune cells patrol the luminal surface of alveoli [171]. Alveolar macrophages are implicated in the development of COPD, and show impaired phagocytosis of pathogens and efferocytosis of apoptotic cells, a feature that might contribute to worsening inflammation [171,214]. Smoking also changes miRNA expression in alveolar macrophages [189]. In alveolar macrophages from current and never smokers, 43 miRNAs are downregulated in smokers. One of these miRNAs is miR-452. Mechanistically, inhibition of miR-452 induces the expression of MMP-12 [189], a protease that is upregulated in alveolar macrophages from smokers [189,215].

In addition to miRNA playing fundamental roles in inflammation, many miRNAs are also implicated in regulating cell survival. Both miR-23a and miR-421, for example, repress caspase-9 and caspase-3, respectively [216,217]. miR-421 is downregulated in response to smoke [191], whereas the activity of caspase-3 is increased by smoke exposure [218]. This raises the possibility that downregulation of miR-421 in response to smoke may upregulate apoptosis.

#### 3.3.2. Interplay of RBPs with miRNAs

miRNAs and RBPs interact to further fine-tune post-transcriptional regulatory mechanisms. For example, hnRNP K increases *PTGS2* mRNA stability by reducing miR-16 binding to the 3′UTR of *PTGS2* mRNA [219], and overexpression of HuR suppresses the ability of miR-16 to promote *PTGS2* mRNA decay [220]. HuR also promotes the translation of *STAT3* mRNA in myotubes exposed to IFN-γ and TNF-α by binding to its 3′UTR—this binding interferes with miR-330–mediated translation inhibition [221]. HuR could also work cooperatively with miRNA to downregulate gene expression. An example of this is the ability of HuR to promote the interaction of let-7- loaded RISC with the 3′UTR of the proto-oncogene *MYC* mRNA to repress its expression [222]. Other examples include the ability of the RBP Pumilio-1 to bind to 3′UTR of p27 (*CDKN1B*; cyclin dependent kinase inhibitor 1b) mRNA, and facilitate its association with miR-221/222 to destabilize p27 expression [223]. While these examples are outside the context of COPD, the discovery of crosstalk between RBPs and miRNAs provides support for their dynamic regulation of gene expression associated with cellular mechanisms whose dysregulation contributes to the pathogenesis of chronic lung disease development.

### 3.4. LncRNA in COPD

Altered expression of lncRNA is now thought to be involved in the pathogenesis of COPD. For example, a previous study found that 109 lncRNA are differentially expressed in the lungs of mice exposed to CS, of which 51 lncRNAs were significantly upregulated, and 58 were significantly downregulated. Gene ontology analysis of potential lncRNA target protein-coding genes showed enrichment in pathways involved in the cellular response to interferon-beta [224]. Furthermore, genome-wide expression analysis of lncRNAs in lung tissue from non-smokers and smokers with/without COPD showed differential expression of hundreds of lncRNAs in COPD, independent of smoking [225]. Another study found that 8376 lncRNAs are differentially expressed in COPD lung, of which 3939 are upregulated, and 4437 are downregulated [226]. However, the mechanism of how lncRNA affect the pathogenesis of COPD is not fully understood.

#### 3.4.1. LncRNA and Pathogenic Mechanisms of COPD

Dysregulation of lncRNA expression suggests they have roles in the pathogenesis of CS-related diseases. For example, lncRNA are upregulated by smoking: this includes smoke- and cancer–associated lncRNA–1 (SCAL1) [227], and lung cancer progression–association transcript 1 (LCPAT1) [228]. LCPAT1 is involved in smoke-induced DNA damage [228], and SCAL1 protects against CS–induced toxicity [227], suggesting that SCAL1 serves as a protective mechanism against smoke. Metastasis-associated in lung adenocarcinoma transcript 1 (MALAT1) [229] and HOX transcript antisense RNA (HOTAIR) [230] are also upregulated in response to CS, and involved in CS-induced EMT.

LncRNAs that are involved in EMT are also increased in COPD, including MALAT1 [231] and taurine-upregulated gene 1 (TUG1) [226]. The knockdown of MALAT1 in human lung fibroblasts reduces the expression of fibronectin and α-smooth muscle actin, proteins that are involved in fibrogenesis, in response to transforming growth factor β (TGF-β) [231]. The knockdown of TUG1 also decreases the expression of fibronectin and α-smooth muscle actin in human lung epithelial cells and lung fibroblasts exposed to TGF-β [226]. These data suggest that lncRNAs may regulate fibrosis formation around the small airways in COPD, in part, via the regulation of EMT.

LncRNAs are also involved in inflammation, such as maternally expressed gene 3 (MEG3) [232], MALAT1 [233], and HOTAIR [234]. Silencing of MEG3 inhibits apoptosis, and reduces inflammatory mediators in human bronchial epithelial cells exposed to CSE [232]. Interestingly, MEG3 is elevated in blood samples from COPD patients and smokers [232], suggesting that MEG3 may regulate CS-induced inflammation and apoptosis. Additionally, in macrophages exposed to LPS, the expression of MALAT1 is upregulated, which, in turn, interacts with nuclear factor kappa B (NF-κB) in the nucleus, and reduces the expression of inflammatory cytokines [233]. The expression of HOTAIR is also upregulated in in cardiomyocytes exposed to LPS. Conversely, HOTAIR induces inflammatory response by activating NF-κB [234], and regulates oxidative stress and apoptosis [235]. It is currently not known whether the increase in MALAT1 and HOTAIR expression from CS [229,230] regulates inflammation and/or apoptosis in COPD pathogenesis.

#### 3.4.2. Interplay of RBPs with lncRNA

LncRNA are known to interact with RBPs, a feature which might be important in understanding the functional role of RBPs and lncRNAs in the development of lung diseases. For example, the lncRNA c-Myc-upregulated (MYU) associates with hnRNP K to stabilize the mRNA of cyclin-dependent kinase 6 (*CDK6*), which promotes G1/S transition of the cell cycle. HnRNP K also inhibits miR-16 binding to the 3′UTR of *CDK6* [236]. Although hnRNP K stabilizes *PTGS2* mRNA by reducing the association of miR-16 to the *PTGS2* 3′UTR [219], hnRNP K may collaborate with MYU to regulate the stability of *PTGS2* mRNA. Another example of such an association is the lncRNA functional intergenic repeating RNA element (FIRRE), which interacts with hnRNP U to stabilize vascular cell adhesion molecule 1 (VCAM1), a cell adhesion molecule that is involved in inflammation [237]. Finally, the lncRNA lincRNA regulator of reprogramming (Linc-RoR) stabilizes the proto-oncogene *MYC* mRNA by interacting with two RBPs. Linc-RoR interacts with hnRNP I to stabilize *MYC* mRNA, and interacts with the destabilizing RBP AUF1 to inhibit its binding to *MYC* mRNA [238]. This suggests that Linc-RoR may control the competition of the two RBPs for *MYC* mRNA. The discovery of the interplay between RBPs and lncRNAs highlights their regulation of post-transcriptional mechanisms of target mRNAs. This would be another step in understanding the landscape of genes that are involved in the pathogenesis of chronic lung disease.

## 4. Conclusions

RBPs, miRNA, and lncRNA are examples of post-transcriptional regulons that may be involved in COPD pathogenesis. To date, many studies have largely focused on transcriptional regulatory pathways implicated in the development of COPD. However, it is increasingly apparent that post-transcriptional regulation of gene expression adds a dynamic layer of complexity to chronic diseases, as RBPs regulate polyadenylation, pre-mRNA splicing, RNA modification, nuclear export, localization, and turnover of target mRNAs. Similarly, miRNA and lncRNA typically regulate post-transcriptional repression of target mRNAs. RBPs, miRNA, and lncRNA function independently, or may operate cooperatively or competitively, adding complexity to the system. Altered function of post-transcriptional regulation may contribute to the development of chronic diseases, particularly those caused by environmental exposures, such as COPD, and future work should address these mechanisms. In this regard, both RBPs and miRNAs represent potential therapeutic targets. For instance, MS-444 is a small molecule inhibitor that interferes with the RNA binding activity of HuR [239], and has been shown to exhibit antitumor activity in vitro and in vivo [240]. miRNAs are also targeted as disease treatment, such as *let-7*, whose exogenous delivery reduces tumor development in a mouse model of lung cancer [241]. Therefore, post-transcriptional regulation of protein expression deserves serious consideration in therapeutic strategies for smoke-related diseases, such as COPD.

## Abbreviations

α1AT	Alpha 1-anti-trypsin
ADAR	Adenosine deaminases acting on RNA
AhR	Aryl hydrocarbon receptor
AUF1	AU-binding factor 1
ARE	AU-rich elements
AS	Alternative splicing
BAL	Bronchoalveolar lavage
CCL	C-C motif ligand
COPD	Chronic obstructive pulmonary disease
COX-2/PTGS2	Cyclooxygenase-2
CS	Cigarette smoke
CSE	Cigarette smoke extract
CXCL	C-X-C motif ligand
EMT	Epithelial-to-mesenchymal transition
GOLD	Global initiative for chronic obstructive lung disease
GR	Glucocorticoid receptors
hnRNP	Heterogeneous nuclear ribonucleoprotein
HOTAIR	HOX transcript antisense RNA
HuR	Hu antigen R
IL	Interleukin
IRP2	Iron-responsive element binding protein 2
LCPAT1	Lung cancer progression–association transcript 1
Linc-RoR	LincRNA regulator of reprogramming
LncRNA	Long non-coding RNAs
LPS	Lipopolysaccharide
MALAT1	Metastasis-associated in lung adenocarcinoma transcript 1
MEG3	Maternally expressed gene 3
MiRNA/MiR	MicroRNA
MMP	Matrix metalloproteinase
NE	Neutrophil Elastase
NF-κB	Nuclear Factor Kappa B
PABP	Poly(A)-binding protein
RBD	RNA-binding domain
RBM5	RNA-binding motif protein 5
RBP	RNA-binding protein
RISC	RNA-induced silencing complex
RNP	Ribonucleoprotein
SCAL1	Smoke and cancer–associated lncRNA–1
SGs	Stress granules
TIA-1	T-cell Intracellular Antigen 1
TNF-α	Tumor necrosis factor α
TTP	Tristetraprolin
UTR	Untranslated region
VEGF	Vascular endothelial growth factor
